# Supplementary material for: Miniaturised implantable circular polarized antenna with a high ARBW
Source: PLoS One. 2025 May 7;20(5):e0321670. doi: 10.1371/journal.pone.0321670 (PMC12057919; doi:10.1371/journal.pone.0321670)
Supplement: S1 File — (PDF) [file pone.0321670.s001.pdf]

Data file of proposed research work

Reflection Coefficient Data		Axial ratio bandwidth Data		Directive Normalised Gain Data			Parameteric study of Sorting pin p		
Freq [GHz]	dB(S(1,1)) []	Freq [GHz]	dB(AxialRatioValue) [] - Phi='0deg' Theta='0deg'	Theta [deg]	dB(GainTotal) [] - Freq='2.45 GHz' Phi='0deg'	dB(GainTotal) [] - Freq='2.45 GHz' Phi='90deg'	Freq [GHz]	S 11 of Position (0.5, 0.25, 0)	S 11 of Position (0.5, 0.3, 0)
2	-1.355420208	2	3.079937425	-180	-9.785878864	-9.665874184	2	-5.680074263	-0.575540032
2.01	-1.364842333	2.01	2.954341376	-178	-9.709831869	-9.609560485	2.01	-6.218431573	-0.590540201
2.02	-1.377066497	2.02	2.832097794	-176	-9.739792222	-9.650842785	2.02	-6.834782712	-0.606655286
2.03	-1.392200588	2.03	2.713174712	-174	-9.894931079	-9.802168366	2.03	-7.543105151	-0.623964179
2.04	-1.41037482	2.04	2.597556303	-172	-10.19723495	-10.07602839	2.04	-8.360543787	-0.642553352
2.05	-1.431743644	2.05	2.48524183	-170	-10.67064161	-10.48334106	2.05	-9.308149793	-0.662517667
2.06	-1.456488034	2.06	2.376243471	-168	-11.339307	-11.0304179	2.06	-10.41137818	-0.683961264
2.07	-1.484818213	2.07	2.270585431	-166	-12.22289787	-11.71302675	2.07	-11.69909802	-0.706998587
2.08	-1.516976881	2.08	2.168301561	-164	-13.32317725	-12.5050786	2.08	-13.19670423	-0.731755486
2.09	-1.553243015	2.09	2.069434005	-162	-14.58805543	-13.33974935	2.09	-14.89841847	-0.75837056
2.1	-1.593936337	2.1	1.974030638	-160	-15.83277297	-14.08864925	2.1	-16.67539254	-0.786996426
2.11	-1.639422567	2.11	1.882143158	-158	-16.65959524	-14.5698832	2.11	-18.06808032	-0.817801489
2.12	-1.690119584	2.12	1.793824468	-156	-16.64510645	-14.63254231	2.12	-18.29341297	-0.850971557
2.13	-1.746504662	2.13	1.709126683	-154	-15.83306627	-14.27610606	2.13	-17.19234866	-0.886711943
2.14	-1.80912298	2.14	1.628098775	-152	-14.67365945	-13.6515747	2.14	-15.4972255	-0.925249603
2.15	-1.878597635	2.15	1.550784188	-150	-13.53400757	-12.94268701	2.15	-13.79106376	-0.966835685
2.16	-1.955641439	2.16	1.477218772	-148	-12.57278991	-12.28038172	2.16	-12.26979838	-1.011748195
2.17	-2.041070865	2.17	1.40742952	-146	-11.83076143	-11.7339879	2.17	-10.95940419	-1.060295112
2.18	-2.135822543	2.18	1.341432425	-144	-11.30261681	-11.33184912	2.18	-9.838479549	-1.112817686
2.19	-2.240972851	2.19	1.27923255	-142	-10.96819281	-11.08007857	2.19	-8.877759854	-1.16969419
2.2	-2.357761228	2.2	1.220823385	-140	-10.80372822	-10.97359744	2.2	-8.050528758	-1.231343837
2.21	-2.487618017	2.21	1.166187248	-138	-10.7854556	-11.00159418	2.21	-7.33455232	-1.298231129
2.22	-2.632197852	2.22	1.11529661	-136	-10.89048328	-11.14989371	2.22	-6.711780286	-1.370870416
2.23	-2.793419864	2.23	1.068115577	-134	-11.09682037	-11.40178599	2.23	-6.167600333	-1.449830611
2.24	-2.973516371	2.24	1.024600917	-132	-11.3832383	-11.73817484	2.24	-5.690124613	-1.535740006
2.25	-3.175092194	2.25	0.984704622	-130	-11.72921121	-12.13755135	2.25	-5.2696095	-1.6292907
2.26	-3.401197515	2.26	0.948374536	-128	-12.11493324	-12.57611295	2.26	-4.898006619	-1.731242805
2.27	-3.655418192	2.27	0.915554273	-126	-12.5211884	-13.02818011	2.27	-4.568619223	-1.842427456
2.28	-3.941989032	2.28	0.886182623	-124	-12.92861949	-13.46681773	2.28	-4.275838693	-1.963747979
2.29	-4.265937904	2.29	0.860190903	-122	-13.31583428	-13.8643135	2.29	-4.014940565	-2.096178653
2.3	-4.633272225	2.3	0.837498502	-120	-13.65611914	-14.1922047	2.3	-3.78192507	-2.240758859
2.31	-5.05122533	2.31	0.81800695	-118	-13.9137715	-14.42122547	2.31	-3.573390869	-2.398581233
2.32	-5.528589791	2.32	0.801594413	-116	-14.04337436	-14.52268214	2.32	-3.386434258	-2.570771035
2.33	-6.076180911	2.33	0.788107271	-114	-13.99717532	-14.47308998	2.33	-3.218567719	-2.7584521
2.34	-6.707501113	2.34	0.777354529	-112	-13.74259385	-14.261806	2.34	-3.067653743	-2.962695638
2.35	-7.439724254	2.35	0.769102954	-110	-13.28126559	-13.89754673	2.35	-2.931850533	-3.184444163
2.36	-8.295205574	2.36	0.763073251	-108	-12.65363827	-13.40843373	2.36	-2.809567356	-3.42440311
2.37	-9.303881504	2.37	0.758940896	-106	-11.92379381	-12.83451129	2.37	-2.699427399	-3.682888346

2.38	-10.50721224	2.38	0.7563373	-104	-11.15700914	-12.21729593	2.38	-2.600236999	-3.959620547
2.39	-11.96479987	2.39	0.754855465	-102	-10.40447959	-11.59180117	2.39	-2.510959824	-4.253454286
2.4	-13.76521575	2.4	0.754054884	-100	-9.698701485	-10.98317353	2.4	-2.43069532	-4.562036328
2.41	-16.03932486	2.41	0.75347069	-98	-9.055537662	-10.40698743	2.41	-2.358660549	-4.881400939
2.42	-18.93428349	2.42	0.752620132	-96	-8.478617865	-9.871323693	2.42	-2.294174918	-5.20553558
2.43	-22.15460636	2.43	0.75101176	-94	-7.963814452	-9.379192959	2.43	-2.236647305	-5.525995602
2.44	-22.99189026	2.44	0.748151137	-92	-7.503059227	-8.930549225	2.44	-2.185565197	-5.831712395
2.45	-20.19010695	2.45	0.743548469	-90	-7.087345327	-8.523625824	2.45	-2.140485509	-6.109206122
2.46	-17.09436345	2.46	0.736723853	-88	-6.708844031	-8.155602407	2.46	-2.101026879	-6.343439989
2.47	-14.62533434	2.47	0.72721148	-86	-6.362061138	-7.822759445	2.47	-2.06686315	-6.519457638
2.48	-12.68744492	2.48	0.71456402	-84	-6.044044997	-7.520352748	2.48	-2.03771794	-6.624667904
2.49	-11.13397772	2.49	0.69835464	-82	-5.753832146	-7.242466355	2.49	-2.013360115	-6.651240652
2.5	-9.861754532	2.5	0.678180384	-80	-5.491468884	-6.982080927	2.5	-1.99360005	-6.597792484
2.51	-8.801803001	2.51	0.653662936	-78	-5.256984366	-6.731518993	2.51	-1.978286614	-6.469669303
2.52	-7.906836838	2.52	0.6244501	-76	-5.049605679	-6.483289602	2.52	-1.967304766	-6.277704817
2.53	-7.143230803	2.53	0.590216692	-74	-4.867359682	-6.231168534	2.53	-1.960573722	-6.035988693
2.54	-6.486232462	2.54	0.550665365	-72	-4.707068893	-5.971182159	2.54	-1.958045648	-5.759477079
2.55	-5.917071552	2.55	0.505527475	-70	-4.564654317	-5.702118371	2.55	-1.959704841	-5.462106552
2.56	-5.421159981	2.56	0.454563927	-68	-4.435609505	-5.425335598	2.56	-1.96556737	-5.155680467
2.57	-4.98693084	2.57	0.397568628	-66	-4.315498233	-5.143928958	2.57	-1.975681177	-4.849459626
2.58	-4.605063008	2.58	0.334373986	-64	-4.200346155	-4.861579599	2.58	-1.990126623	-4.550234714
2.59	-4.267945956	2.59	0.264868114	-62	-4.086840402	-4.581508107	2.59	-2.009017497	-4.262658276
2.6	-3.969299005	2.6	0.189052503	-60	-3.972312933	-4.305846764	2.6	-2.032502498	-3.989661707
2.61	-3.703892978	2.61	0.107382258	-58	-3.85454934	-4.035526655	2.61	-2.06076722	-3.732867181
2.62	-3.467341781	2.62	0.028504423	-56	-3.731517438	-3.770565853	2.62	-2.094036665	-3.492941843
2.63	-3.255943059	2.63	0.084642637	-54	-3.60113506	-3.51052896	2.63	-2.132578353	-3.269884402
2.64	-3.0665542	2.64	0.185515336	-52	-3.461186909	-3.254933792	2.64	-2.176706074	-3.063246599
2.65	-2.896494376	2.65	0.294260015	-50	-3.309456301	-3.003482704	2.65	-2.22678435	-2.872295114
2.66	-2.743466182	2.66	0.409659751	-48	-3.144066797	-2.756129524	2.66	-2.283233694	-2.696131021
2.67	-2.605492295	2.67	0.531290298	-46	-2.963948357	-2.513084672	2.67	-2.346536789	-2.533772059
2.68	-2.480863813	2.68	0.658780522	-44	-2.769280778	-2.274866403	2.68	-2.417245679	-2.384211008
2.69	-2.368097837	2.69	0.791713008	-42	-2.561754164	-2.042434526	2.69	-2.495990109	-2.246452893
2.7	-2.265902438	2.7	0.929596532	-40	-2.344536407	-1.817347672	2.7	-2.583487199	-2.119539668
2.71	-2.17314761	2.71	1.07185599	-38	-2.121933954	-1.601828352	2.71	-2.680552584	-2.002565075
2.72	-2.088841125	2.72	1.217828466	-36	-1.898831218	-1.398635229	2.72	-2.788113279	-1.894681847
2.73	-2.012108468	2.73	1.366762144	-34	-1.680052839	-1.210717471	2.73	-2.907222426	-1.795105794
2.74	-1.942176178	2.74	1.517820438	-32	-1.469794806	-1.040716924	2.74	-3.039076152	-1.703115526
2.75	-1.878358099	2.75	1.670088406	-30	-1.271229352	-0.890444133	2.75	-3.185032889	-1.618050808
2.76	-1.820044106	2.76	1.822581733	-28	-1.086332007	-0.760463534	2.76	-3.346635225	-1.539309519
2.77	-1.766690969	2.77	1.974260011	-26	-0.915929639	-0.649890335	2.77	-3.525634592	-1.466344044
2.78	-1.717815041	2.78	2.12404237	-24	-0.759934083	-0.556449196	2.78	-3.724018851	-1.398657208
2.79	-1.672986486	2.79	2.270825427	-22	-0.617705365	-0.476791693	2.79	-3.944042687	-1.33579807
2.8	-1.631824766	2.8	2.413503597	-20	-0.488477288	-0.407023778	2.8	-4.188260572	-1.277358235
2.81	-1.593995113	2.81	2.55099056	-18	-0.371773936	-0.343357808	2.81	-4.459560938	-1.222967699
2.82	-1.55920569	2.82	2.682239336	-16	-0.267749538	-0.282777884	2.82	-4.761200039	-1.172291339
2.83	-1.527205172	2.83	2.806265349	-14	-0.177397455	-0.223598055	2.83	-5.096831176	-1.125025646
2.84	-1.497780497	2.84	2.922162934	-12	-0.102596186	-0.165806993	2.84	-5.470522173	-1.080895537

2.85	-1.470754558	2.85	3.029124125	-10	-0.045987202	-0.111131797	2.85	-5.886747406	-1.039651659
2.86	-1.4459837	2.86	3.126450159	-8	-0.010705144	-0.062809651	2.86	-6.350329846	-1.001067787
2.87	-1.423354943	2.87	3.213562667	-6	4.38E-09	-0.025113032	2.87	-6.86628704	-0.964938546
2.88	-1.402782915	2.88	3.2900072	-4	-0.016800919	-0.002715318	2.88	-7.439496654	-0.931077288
2.89	-1.384206594	2.89	3.355455637	-2	-0.063273048	-1.17E-09	2.89	-8.074026064	-0.899314357
2.9	-1.367585962	2.9	3.409704074	0	-0.140414941	-0.020410261	2.9	-8.77184251	-0.869495272
2.91	-1.352898729	2.91	3.452666287	2	-0.247738922	-0.065916445	2.91	-9.53041171	-0.841479325
2.92	-1.340137288	2.92	3.484366213	4	-0.383071728	-0.136657994	2.92	-10.33840769	-0.815138186
2.93	-1.329306036	2.93	3.504927775	6	-0.542508984	-0.230800077	2.93	-11.1686269	-0.790354715
2.94	-1.320419147	2.94	3.514565038	8	-0.72055216	-0.344632885	2.94	-11.96814423	-0.767021923
2.95	-1.313498871	2.95	3.513571714	10	-0.91044709	-0.472926407	2.95	-12.65006544	-0.745041922
2.96	-1.308574367	2.96	3.502309852	12	-1.104724762	-0.609527012	2.96	-13.10047341	-0.724325147
2.97	-1.305681035	2.97	3.481204636	14	-1.295915628	-0.748139706	2.97	-13.21761817	-0.704789533
2.98	-1.304860334	2.98	3.450734864	16	-1.477369931	-0.883187962	2.98	-12.97073151	-0.686359811
2.99	-1.306159986	2.99	3.411429455	18	-1.644076431	-1.010600303	2.99	-12.42168899	-0.668966933
3	-1.309634542	3	3.363864633	20	-1.793343871	-1.128364042	3	-11.68320793	-0.652547458
				22	-1.925209159	-1.236726483			
				24	-2.042473902	-1.338004417			
				26	-2.150342857	-1.436054709			
				28	-2.255723511	-1.535528993			
				30	-2.366315084	-1.641066003			
				32	-2.489644268	-1.756567809			
				34	-2.632190136	-1.884674297			
				36	-2.798697268	-2.026505314			
				38	-2.991727244	-2.181687573			
				40	-3.211460923	-2.348626871			
				42	-3.455741937	-2.524934567			
				44	-3.72033966	-2.707889063			
				46	-3.999398234	-2.894828142			
				48	-4.286020582	-3.083429564			
				50	-4.572914808	-3.271916468			
				52	-4.853015709	-3.459268537			
				54	-5.120000773	-3.645487372			
				56	-5.368655856	-3.831862706			
				58	-5.595102587	-4.021080952			
				60	-5.796952042	-4.216995076			
				62	-5.973466062	-4.423974953			
				64	-6.125770385	-4.645937649			
				66	-6.257084385	-4.885322597			
				68	-6.372853575	-5.142349553			
				70	-6.480646369	-5.414855913			
				72	-6.589732006	-5.698871526			
				74	-6.710367433	-5.989877789			
				76	-6.852925962	-6.284451164			
				78	-7.027046427	-6.581794694			
				80	-7.240957582	-6.88463705			
				82	-7.501067217	-7.199192752			

				84	-7.811836949	-7.53423969			
				86	-8.17591356	-7.899673293			
				88	-8.594455414	-8.30498005			
				90	-9.067564128	-8.757940014			
				92	-9.594694429	-9.263641563			
				94	-10.17486754	-9.82369583			
				96	-10.80647048	-10.43543727			
				98	-11.48641324	-11.09092047			
				100	-12.20847343	-11.77572014			
				102	-12.96082702	-12.46798709			
				104	-13.72311121	-13.13889386			
				106	-14.46396292	-13.75610279			
				108	-15.14074541	-14.29114096			
				110	-15.70353099	-14.72869255			
				112	-16.10417527	-15.0724583			
				114	-16.30806184	-15.34267606			
				116	-16.30326698	-15.5658502			
				118	-16.10313744	-15.76239505			
				120	-15.74259261	-15.93817612			
				122	-15.27097411	-16.08321148			
				124	-14.74387419	-16.17827588			
				126	-14.21569968	-16.20739444			
				128	-13.73445108	-16.17025531			
				130	-13.33939939	-16.08684686			
				132	-13.06124068	-15.9914141			
				134	-12.92369907	-15.92043168			
				136	-12.94556485	-15.90161078			
				138	-13.14240619	-15.94735404			
				140	-13.5272872	-16.05219344			
				142	-14.10946155	-16.19276906			
				144	-14.88876398	-16.33006513			
				146	-15.84061927	-16.41508969			
				148	-16.88285242	-16.39919441			
				150	-17.82289548	-16.24743236			
				152	-18.34616246	-15.94912015			
				154	-18.19491046	-15.51928592			
				156	-17.43366663	-14.99056885			
				158	-16.36608088	-14.40151092			
				160	-15.2544697	-13.78747378			
				162	-14.22961412	-13.1763483			
				164	-13.33361382	-12.58797868			
				166	-12.56766742	-12.03546625			
				168	-11.91812125	-11.52706528			
				170	-11.36835676	-11.06804917			
				172	-10.90400641	-10.66232226			
				174	-10.51522257	-10.31371689			
				176	-10.19758179	-10.02696169			

				178	-9.952276545	-9.808315993			
				180	-9.785878864	-9.665874184			

ositions					Parametric study of metamaterials		
S 11 of Position (0.25, 5.75, 0)	S 11 of Position (3, 2.25, 0)	S 11 of Position (3, 5.75, 0)	S 11 of Position (3.25, 0.25, 0)	S 11 of Position (6, 0.25, 0)	Freq [GHz]	dB(AxialRatio Value) of one metamaterial - Phi='0deg' Theta='0deg'	dB(AxialRatioValue) of Two diagnal metamaterial - Phi='0deg' Theta='0deg'
-1.877594629	-0.302434848	-15.77428022	-1.929430209	-1.455877996	2	3.523800731	3.079937425
-2.007557654	-0.308752474	-14.18508234	-1.998289168	-1.462396758	2.01	3.432968987	2.954341376
-2.150698581	-0.31523246	-12.65953092	-2.074860966	-1.471635755	2.02	3.345734709	2.832097794
-2.308512785	-0.321881537	-11.30371061	-2.159946915	-1.48367486	2.03	3.261957132	2.713174712
-2.482654884	-0.328706836	-10.12710487	-2.254465523	-1.498613097	2.04	3.181489194	2.597556303
-2.67494151	-0.335715888	-9.111232647	-2.359471151	-1.516569971	2.05	3.104175127	2.48524183
-2.887346754	-0.342916598	-8.232760751	-2.476176188	-1.537687081	2.06	3.02985038	2.376243471
-3.121984474	-0.350317195	-7.470179366	-2.605977484	-1.56213005	2.07	2.958341031	2.270585431
-3.381067998	-0.357926143	-6.805240803	-2.750488018	-1.590090824	2.08	2.889462594	2.168301561
-3.666840221	-0.365752007	-6.222854615	-2.911574989	-1.621790369	2.09	2.823020489	2.069434005
-3.981452879	-0.373803244	-5.71060852	-3.091405922	-1.65748185	2.1	2.758810225	1.974030638
-4.326774325	-0.382087926	-5.258269139	-3.292504856	-1.697454338	2.11	2.696618031	1.882143158
-4.704089769	-0.390613343	-4.857355348	-3.517821438	-1.742037144	2.12	2.636221719	1.793824468
-5.113648659	-0.399385513	-4.500795635	-3.770816741	-1.791604886	2.13	2.577392694	1.709126683
-5.554000522	-0.408408553	-4.182658376	-4.055571283	-1.846583386	2.14	2.519897831	1.628098775
-6.021055754	-0.417683949	-3.89794012	-4.376923097	-1.907456571	2.15	2.463501465	1.550784188
-6.506841498	-0.427209742	-3.642398689	-4.740647592	-1.974774537	2.16	2.40796846	1.477218772
-6.998012327	-0.436979712	-3.412420611	-5.153697149	-2.04916298	2.17	2.353067164	1.40742952
-7.474432474	-0.446982671	-3.204914925	-5.624528755	-2.131334265	2.18	2.298572074	1.341432425
-7.908575152	-0.457202023	-3.017227411	-6.163565687	-2.222100426	2.19	2.244267154	1.27923255
-8.266990684	-0.467615802	-2.847070758	-6.783870672	-2.322388484	2.2	2.189949283	1.220823385
-8.515132409	-0.478197355	-2.692467321	-7.502165787	-2.433258539	2.21	2.135430562	1.166187248
-8.625466375	-0.488916824	-2.551701889	-8.340446325	-2.555925209	2.22	2.080540915	1.11529661
-8.586012439	-0.499743399	-2.42328251	-9.328665519	-2.691783134	2.23	2.025130964	1.068115577
-8.404540196	-0.510648188	-2.305907822	-10.50947405	-2.842437456	2.24	1.969072393	1.024600917
-8.105624364	-0.521607295	-2.198439706	-11.94722529	-3.009740418	2.25	1.912260024	0.984704622
-7.722569546	-0.532604619	-2.099880294	-13.74679756	-3.1958356	2.26	1.854611655	0.948374536
-7.288953141	-0.543633839	-2.009352562	-16.09842177	-3.403211782	2.27	1.796067099	0.915554273
-6.833151087	-0.554699236	-1.926083914	-19.40679844	-3.634769127	2.28	1.736587637	0.886182623
-6.376351147	-0.565815231	-1.849392237	-24.7874624	-3.893901376	2.29	1.676153162	0.860190903
-5.932880825	-0.577004799	-1.778674027	-34.42637177	-4.184599314	2.3	1.614759232	0.837498502
-5.511535613	-0.588297163	-1.713394249	-25.79428051	-4.511583063	2.31	1.552414028	0.81800695
-5.117070598	-0.599725211	-1.65307766	-19.99816475	-4.880474462	2.32	1.489133166	0.801594413
-4.751479646	-0.611323053	-1.597301361	-16.54330516	-5.298026675	2.33	1.424935308	0.788107271
-4.414965841	-0.623124025	-1.545688388	-14.12692343	-5.772437933	2.34	1.35983694	0.777354529
-4.106631384	-0.635159244	-1.497902194	-12.29585406	-6.313792806	2.35	1.293847748	0.769102954
-3.824948463	-0.64745675	-1.453641872	-10.84247771	-6.934703462	2.36	1.22696456	0.763073251
-3.568068769	-0.66004112	-1.41263803	-9.654196086	-7.651276171	2.37	1.159167625	0.758940896

-3.334020036	-0.672933461	-1.374649205	-8.662739748	-8.484629278	2.38	1.090415893	0.7563373
-3.120832076	-0.686151633	-1.339458751	-7.823301938	-9.463392468	2.39	1.020644715	0.754855465
-2.9266077	-0.699710614	-1.306872128	-7.104688496	-10.62805684	2.4	0.949762693	0.754054884
-2.749566191	-0.713622914	-1.276714544	-6.484180636	-12.03907654	2.41	0.877651973	0.75347069
-2.588060764	-0.727898987	-1.248828889	-5.944642611	-13.79330194	2.42	0.804167164	0.752620132
-2.44058649	-0.742547603	-1.223073934	-5.472790126	-16.0612124	2.43	0.72913886	0.75101176
-2.305779038	-0.757576175	-1.199322756	-5.058099274	-19.18408523	2.44	0.65237497	0.748151137
-2.182407505	-0.772991016	-1.177461355	-4.692088361	-23.95559399	2.45	0.573668104	0.743548469
-2.069365571	-0.788797545	-1.157387448	-4.367826884	-30.64701703	2.46	0.492800122	0.736723853
-1.965661617	-0.805000437	-1.139009406	-4.079588512	-25.76210351	2.47	0.409555255	0.72721148
-1.870408592	-0.821603719	-1.122245329	-3.82259863	-20.31537163	2.48	0.323741601	0.71456402
-1.782813736	-0.838610838	-1.107022235	-3.592846054	-16.87264698	2.49	0.235251806	0.69835464
-1.702168288	-0.856024675	-1.093275348	-3.386939533	-14.43642761	2.5	0.144340925	0.678180384
-1.627839127	-0.873847552	-1.08094748	-3.201996401	-12.5822143	2.51	0.054822366	0.653662936
-1.559260566	-0.892081196	-1.0699885	-3.035554811	-11.10660002	2.52	0.061743784	0.6244501
-1.49592703	-0.9107267	-1.060354863	-2.885503687	-9.89755918	2.53	0.159487008	0.590216692
-1.437385702	-0.92978446	-1.052009218	-2.750026179	-8.886761673	2.54	0.265092993	0.550665365
-1.383231798	-0.949254112	-1.04492007	-2.627553628	-8.029260329	2.55	0.375399489	0.505527475
-1.333102072	-0.969134466	-1.039061497	-2.51672778	-7.293741851	2.56	0.490068203	0.454563927
-1.286670773	-0.989423438	-1.03441292	-2.416369583	-6.657397177	2.57	0.609047889	0.397568628
-1.243645258	-1.010118	-1.030958919	-2.325453271	-6.103019715	2.58	0.732335732	0.334373986
-1.203762309	-1.031214143	-1.028689096	-2.243084738	-5.617264275	2.59	0.859929097	0.264868114
-1.16678468	-1.052706863	-1.02759798	-2.168483421	-5.189549522	2.6	0.991810717	0.189052503
-1.132498089	-1.074590174	-1.027684971	-2.100967071	-4.811336058	2.61	1.127943266	0.107382258
-1.100708855	-1.096857162	-1.028954331	-2.039938908	-4.475633585	2.62	1.268264941	0.028504423
-1.071241029	-1.119500056	-1.031415203	-1.984876767	-4.176653268	2.63	1.412685623	0.084642637
-1.043934409	-1.142510349	-1.035081686	-1.935323911	-3.909555334	2.64	1.561080061	0.185515336
-1.018642569	-1.165878919	-1.039972931	-1.890881234	-3.67026114	2.65	1.713279587	0.294260015
-0.995230891	-1.189596182	-1.046113298	-1.851200652	-3.455310172	2.66	1.869061852	0.409659751
-0.973574767	-1.21365222	-1.053532537	-1.815979491	-3.261749178	2.67	2.028137161	0.531290298
-0.953558171	-1.238036895	-1.062266025	-1.784955735	-3.087044883	2.68	2.190135842	0.658780522
-0.935072004	-1.262739914	-1.072355043	-1.757904008	-2.929014367	2.69	2.354592506	0.791713008
-0.91801262	-1.287750839	-1.083847108	-1.734632184	-2.785768951	2.7	2.520933199	0.929596532
-0.902280737	-1.313059014	-1.09679635	-1.714978558	-2.655668605	2.71	2.68846521	1.07185599
-0.887780122	-1.338653431	-1.111263948	-1.698809501	-2.537284663	2.72	2.856371659	1.217828466
-0.874416548	-1.36452252	-1.12731863	-1.68601754	-2.429369179	2.73	3.023712229	1.366762144
-0.862097215	-1.390653881	-1.145037233	-1.676519829	-2.330829656	2.74	3.189433554	1.517820438
-0.850730027	-1.417033979	-1.164505338	-1.670256975	-2.24070814	2.75	3.352387669	1.670088406
-0.840223497	-1.443647826	-1.18581798	-1.667192181	-2.158163852	2.76	3.511361073	1.822581733
-0.830486816	-1.470478664	-1.209080445	-1.667310707	-2.082458713	2.77	3.665111314	1.974260011
-0.821430597	-1.497507674	-1.234409157	-1.670619614	-2.012945172	2.78	3.812410083	2.12404237
-0.812967668	-1.524713726	-1.261932666	-1.677147804	-1.949055886	2.79	3.95209022	2.270825427
-0.805014626	-1.552073176	-1.291792752	-1.686946346	-1.890294837	2.8	4.083091377	2.413503597
-0.79749353	-1.579559717	-1.324145642	-1.700089087	-1.836229563	2.81	4.204501384	2.55099056
-0.790333767	-1.607144283	-1.359163371	-1.71667358	-1.786484232	2.82	4.315590807	2.682239336
-0.783474117	-1.634795009	-1.397035279	-1.73682232	-1.740733377	2.83	4.415835673	2.806265349
-0.776864519	-1.662477226	-1.437969683	-1.760684329	-1.698696143	2.84	4.504929862	2.922162934

-0.770467509	-1.690153509	-1.482195719	-1.788437118	-1.660130997	2.85	4.582786168	3.029124125
-0.764259189	-1.717783753	-1.529965393	-1.820289056	-1.624830842	2.86	4.649525676	3.126450159
-0.758229466	-1.745325278	-1.581555855	-1.856482198	-1.59261857	2.87	4.705459796	3.213562667
-0.752381558	-1.772732966	-1.637271936	-1.897295633	-1.563343054	2.88	4.751065769	3.2900072
-0.746730887	-1.799959417	-1.697448969	-1.943049404	-1.536875613	2.89	4.786957321	3.355455637
-0.741303364	-1.826955127	-1.762455968	-1.994109097	-1.513106972	2.9	4.813856575	3.409704074
-0.736133338	-1.853668694	-1.832699201	-2.050891181	-1.491944705	2.91	4.832561401	3.452666287
-0.731261294	-1.880047031	-1.908626245	-2.113869219	-1.47331117	2.92	4.843917388	3.484366213
-0.726731607	-1.90603561	-1.990730629	-2.183581075	-1.45714189	2.93	4.848789635	3.504927775
-0.722590479	-1.931578721	-2.079557188	-2.260637273	-1.443384346	2.94	4.848037918	3.514565038
-0.718884161	-1.956619747	-2.175708291	-2.345730693	-1.431997132	2.95	4.842490676	3.513571714
-0.715657576	-1.981101468	-2.279851166	-2.439647812	-1.422949427	2.96	4.832924138	3.502309852
-0.712953345	-2.004966366	-2.392726575	-2.543281741	-1.416220724	2.97	4.82004195	3.481204636
-0.71081119	-2.028156964	-2.515159184	-2.657647348	-1.411800788	2.98	4.804454495	3.450734864
-0.709267698	-2.050616164	-2.648070025	-2.783898813	-1.409689795	2.99	4.786663084	3.411429455
-0.708356355	-2.072287605	-2.792491553	-2.923350004	-1.409898624	3	4.767045575	3.363864633

